# Nonredox CO_2_ Fixation in Solvent-Free Conditions Using a Lewis Acid Metal–Organic Framework Constructed from a Sustainably Sourced Ligand

**DOI:** 10.1021/acs.inorgchem.2c02749

**Published:** 2022-11-10

**Authors:** Satarupa Das, Jinfang Zhang, Thomas W. Chamberlain, Guy J. Clarkson, Richard I. Walton

**Affiliations:** † Department of Chemistry, University of Warwick, CoventryCV4 7AL, U.K.; ‡ International Joint Research Center for Photoresponsive Molecules and Materials, School of Chemical and Materials Engineering, Jiangnan University, Wuxi214122, P. R. China

## Abstract

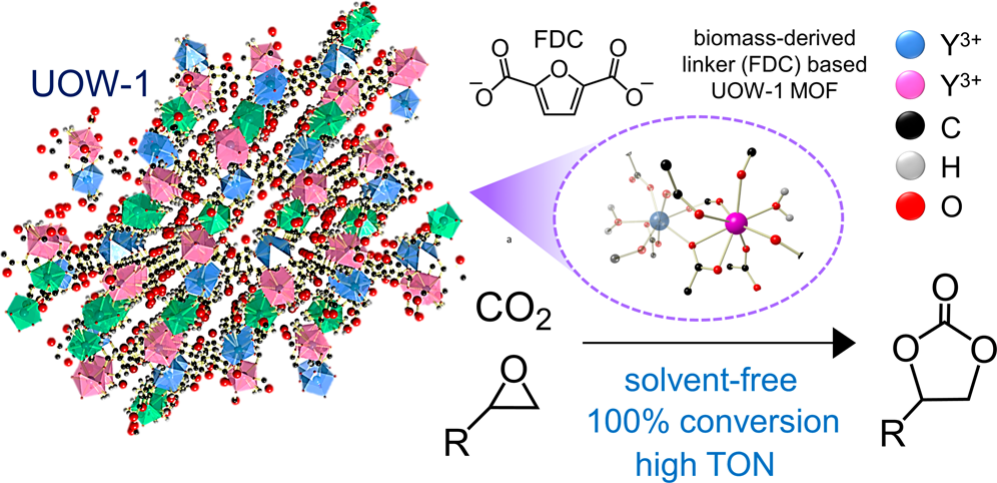

CO_2_ epoxidation to cyclic carbonates under mild, solvent-free conditions is a promising pathway toward sustainable CO_2_ utilization. Metal–organic frameworks (MOFs) explored for such applications so far are commonly composed of nonrenewable ligands such as benzene dicarboxylate (BDC) or synthetically complex linkers and therefore are not suitable for commercial utilization. Here, we report new yttrium 2,5-furandicarboxylate (FDC)-based MOFs: “UOW-1” and “UOW-2” synthesized via solvothermal assembly, with the former having a unique structural topology. The FDC linker can be derived from biomass and is a green and sustainable alternative to conventionally used BDC ligands, which are sourced exclusively from fossil fuels. UOW-1, owing to unique coordination unsaturation and a high density of Lewis active sites, promotes a high catalytic activity (∼100% conversion; ∼99% selectivity), a high turnover frequency (70 h^–1^), and favorable first-order kinetics for CO_2_ epoxidation reactions using an epichlorohydrin model substrate under solvent-free conditions within 6 h and a minimal cocatalyst amount. A systematic catalytic study was carried out by broadening the epoxide substrate scope to determine the influence of electronic and steric factors on CO_2_ epoxidation. Accordingly, higher conversion efficiencies were observed for substrates with high electrophilicity on the carbon center and minimal steric bulk. The work presents the first demonstration of sustainable FDC-based MOFs used for efficient CO_2_ utilization.

## Abstract 2

A metal−organic framework constructed from Y^3+^ and the ligand furan 2,5-dicarboxylate provides Lewis acid sites for the effective ring opening of cyclic peroxide, thus paving the way for effective CO_2_ utilization using a heterogeneous catalyst constructed from a biomass-derived ligand. The material operates under solvent-free conditions and is recyclable.

## Introduction

With increasing anthropogenic CO_2_ emissions contributing to global warming, the demand for effective strategies to mitigate emissions and sustainably convert CO_2_ to value-added products is on the rise.^1,2^ Among various approaches,^3^ 100% atom-economic ring expansion of epoxides via CO_2_ addition yielding cyclic carbonates (CO_2_ cycloaddition or CO_2_ epoxidation) is one of the most appealing, green, and sustainable approaches for CO_2_ utilization.^4^ Despite this promise, the high thermodynamic stability and kinetic inertness of CO_2_ serve as the major bottleneck for CO_2_ epoxidation reactions, for which the development of efficient catalysts is essential.^5^ The presence of active Lewis acid sites in the catalysts along with a nucleophilic species in the reaction medium is a vital prerequisite for effectively catalyzing CO_2_ epoxidation reactions, as indicated in previous studies.^6−8^

Metal–organic frameworks (MOFs), which comprise metal nodes coordinated by polydentate organic ligands, yielding three-dimensional open networks are a class of materials that hold potential for a wide range of applications owing to their structural uniformity, high surface area, tunable porosity, and readily functionalized frameworks.^9,10^ Although several reports exist where MOFs have been explored as heterogeneous catalysts for CO_2_ fixation, including conversion of CO_2_ to cyclic carbonates,^11−13^ the processes are not feasible from the perspective of sustainability. A majority of the MOFs used for such applications typically consist of ligands that are either expensive, toxic, synthesized via complex routes under harsh conditions, obtained from polluting sources, or nonrenewable.^14−17^ For instance, there are MOFs reported with metals such as Cu, Y, etc. and custom-made linker combinations, which involves tedious and harsh multistep synthesis protocols.^14,15^ The development of MOFs with renewable, less toxic ligands obtained from sustainable routes is therefore critical for their applicability in an industrial scale.

Over the years, there has been an increased effort toward the exploration and utilization of biomass as an inexpensive, renewable, and accessible feedstock/precursor for various chemical processes and applications.^18−20^ A range of valuable chemicals can be derived from lignocellulosic biomass. Among these, 2,5-furandicarboxylic acid (H_2_FDC) is an important product that can be produced via selective oxidation of biomass-derived 5-hydroxymethylfurfural (5-HMF).^21^ The use of H_2_FDC (or its deprotonated form: 2,5-furandicarboxylate (FDC)) ligands for synthesizing MOFs is particularly attractive as they are a green and sustainable alternative to conventional nonrenewable ligands used for MOF construction such as benzene dicarboxylates (BDCs; terephthalates and isophthalates), which are sourced from polluting fossil fuels.^22,23^ However, till date, very few FDC-based MOFs have been successfully synthesized and characterized.^24,25^

In this work, we have successfully synthesized two new FDC-based MOFs: UOW-1 and UOW-2 (UOW indicates “University of Warwick”) having chemical formulas of {Y_3_(HFDC)(FDC)_4_(H_2_O)_6_}·3H_2_O and {Y_2_(FDC)_2_(H_2_O)_10_}FDC·6H_2_O, respectively. The choice of yttrium (Y^3+^) as the metal center is primarily due to its characteristic Lewis acidic nature (arising from the *d*-orbital vacancy), which can facilitate catalytic reactions.^26^ Consequently, the MOFs have been applied as catalysts for solvent-free CO_2_ epoxidation reactions to yield cyclic carbonates with high conversion rates and selectivity. UOW-1 emerged as the best catalyst for CO_2_ epoxidation with high turnovers compared to UOW-2 and a Y-centered MOF based on common BDC linkers, which was used for comparison. A detailed kinetic analysis and a systematic study of the influence of substrate properties (electronic and steric effects) provided further insights into the catalytic process.

## Results and Discussion

### Structural Characterization of FDC-Based MOFs

**Figure 1:**
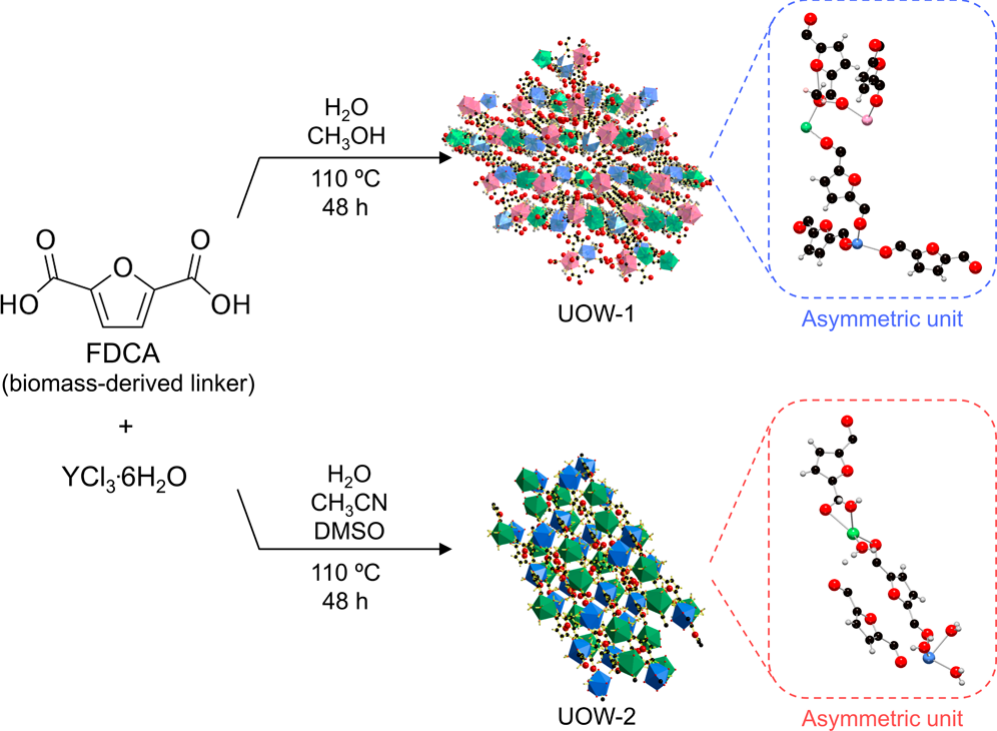
Synthesis scheme and corresponding structures of UOW-1 and UOW-2. Color codes: Y1 - blue, Y2 - green, Y3 - pink, C - black, O - red, and H - gray.

The UOW-1 MOF was synthesized using a solvothermal route in a methanol–water mixture (see the Experimental Section for details, Figure 1). Single-crystal X-ray structural analysis revealed that UOW-1 crystallizes in the monoclinic *P*2_1_/*c* space group and exhibits an unprecedented (3,3,4,4,4,6,6)-c 3D framework (Figure S1 and Table S1).

**Figure 2:**
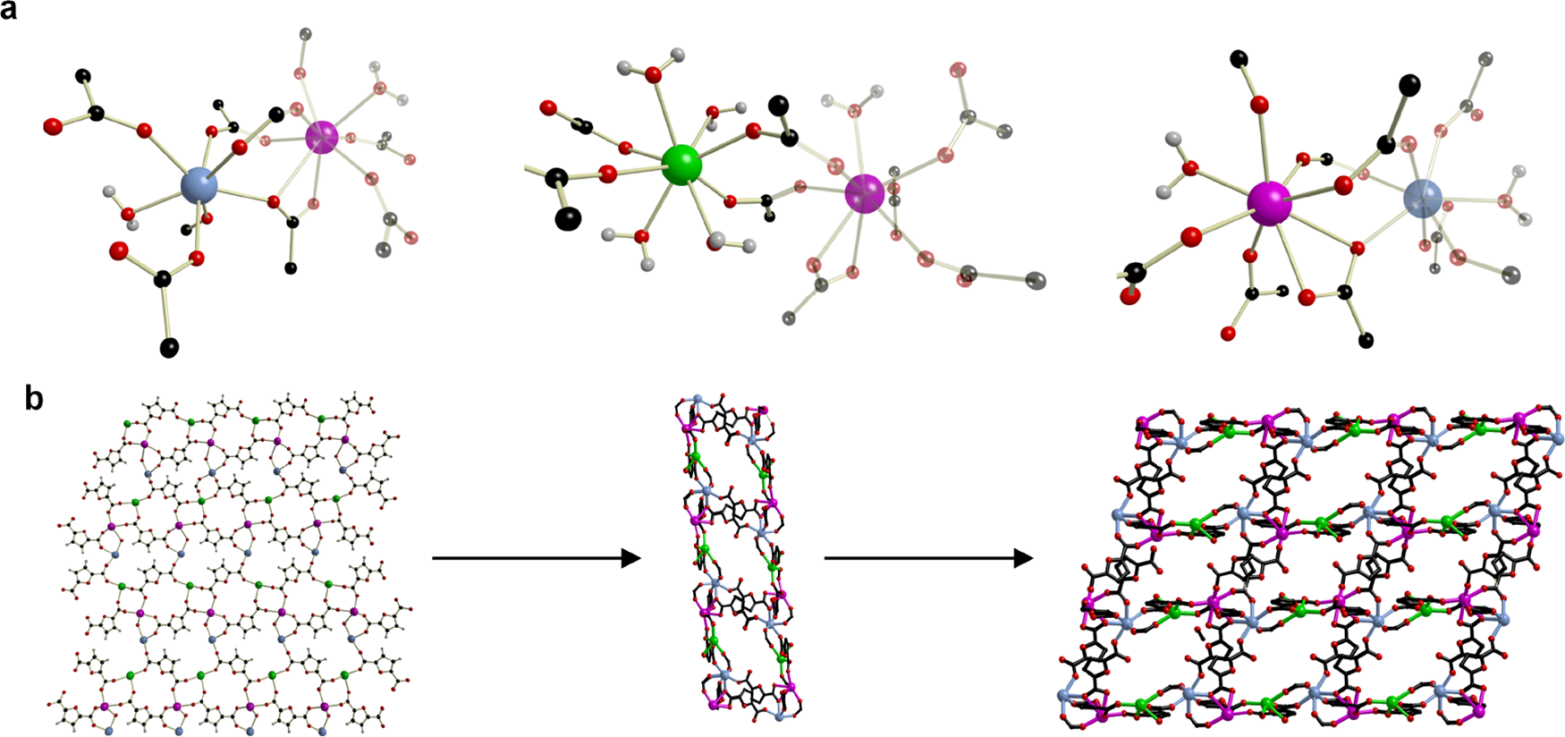
(a) Connectivity and coordination environments of different metal nodes in UOW-1. Color codes: Y1 - blue, Y2 - green, Y3 - pink, C - black, O - red, and H - gray. (b) 2D layers and 3D network formation interlinked via a differently bonded FDC linker.

The asymmetric unit of UOW-1 has three yttrium ions (Y^3+^; seven coordinated Y1, eight coordinated Y2 and Y3), a mono-deprotonated HFDC^–^ ligand (L1), four fully deprotonated FDC^2–^ ligands (L2, L3, L4, and L5), six coordinated H_2_O, and three occluded H_2_O molecules (Figure 2a and Figure S2, details in the Supporting Information).

**Figure 3:**
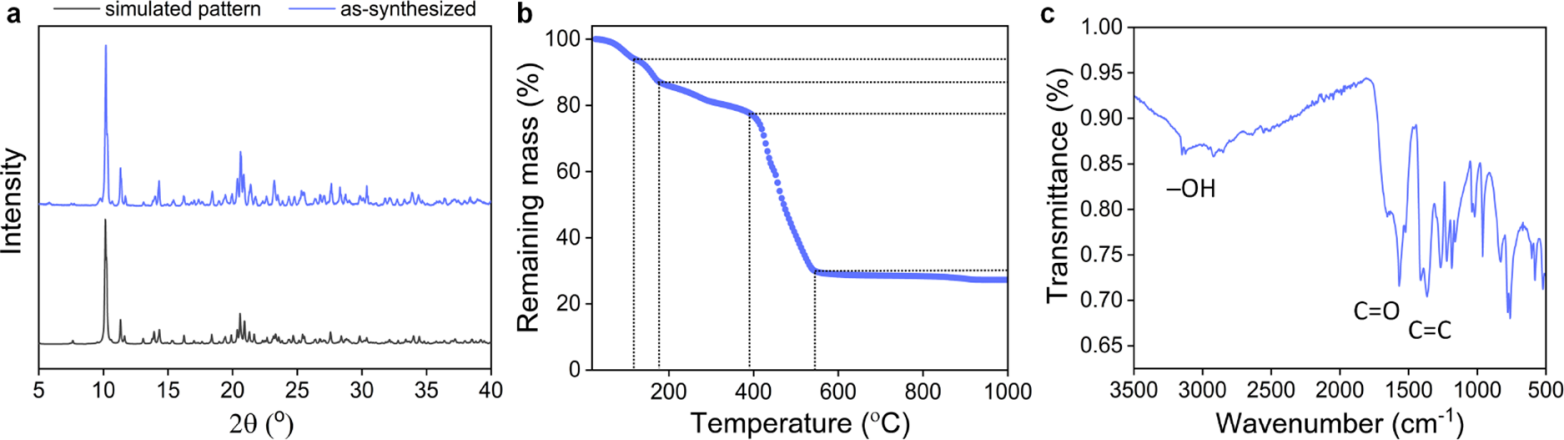
(a) PXRD of as-synthesized UOW-1. (b) TGA of UOW-1 (see Table S2 for assignment) and (c) IR spectra of UOW-1.

Structural analysis indicates that L1, L3, and L4 bridge Y atoms to afford a 2D layer; two 2D layers are supported by L2 to form a more complex 2D double-layer architecture; these unusual double layers are interlinked by L5 to further construct a 3D framework (Figure 2b). The phase purity of UOW-1 was verified by powder X-ray diffraction (PXRD), where the consistency between the diffraction pattern of the synthesized sample and theoretical simulation was confirmed (Figure 3a). Small deviations in Bragg peak intensities are due to the preferred orientation of the polycrystalline sample.

Thermogravimetric analysis (TGA) of UOW-1 revealed the bulk mass loss profile with temperature, which is consistent with that expected from the chemical formula of the MOF, suggesting the phase purity of the sample (Figure 3b and Table S2). The Fourier transform infrared (FTIR) spectrum of UOW-1 showed characteristic broad adsorption peaks between 2500 and 3000 due to hydroxyl stretching of the carboxyl groups and also peaks around 1659 cm^–1^, corresponding to the −C=C and C=O stretches from the allyl and carboxylate, respectively (Figure 3c).^27^

The second newly synthesized MOF UOW-2 (see the Experimental Section for synthesis details) is isostructural to a previously reported lanthanide-based {[Ln_2_(FDC)_2_(H_2_O)_10_]FDC·6H_2_O}_*n*_ MOF (Ln = Dy, Eu, and Gd).^28^ Single-crystal X-ray diffraction revealed that UOW-2 is triclinic with the space group *P*1̅. Its asymmetric unit contains two Y^3+^ ions, two FDC^2–^ anions, 10 coordinated H_2_O molecules, one free FDC^2–^ anion, and six occluded H_2_O molecules (Figure S3 and Table S3). Each yttrium is nine coordinated with the same connectivity. The Y-centered, BDC-based MOF (used in this work for comparing the CO_2_ epoxidation activity to the FDC-based MOFs), Y_6_(BDC)_7_(OH)_4_(H_2_O)_4_ (abbreviated as Y_6_-BDC; see the Experimental Section for synthesis details), has three distinct eight coordinated Y centers bridged via μ^3^-OH groups where four of the yttrium centers are terminally coordinated with water molecules.^29,30^ The Yb version of this MOF has been previously used as a Lewis acid catalyst for the conversion of glucose to HMF.^31^ The phase purity of the as-synthesized UOW-2 and Y_6_-BDC was confirmed using PXRD (Figure S4).

### CO_2_ Epoxidation Using FDC-Based MOFs

**Figure 4:**
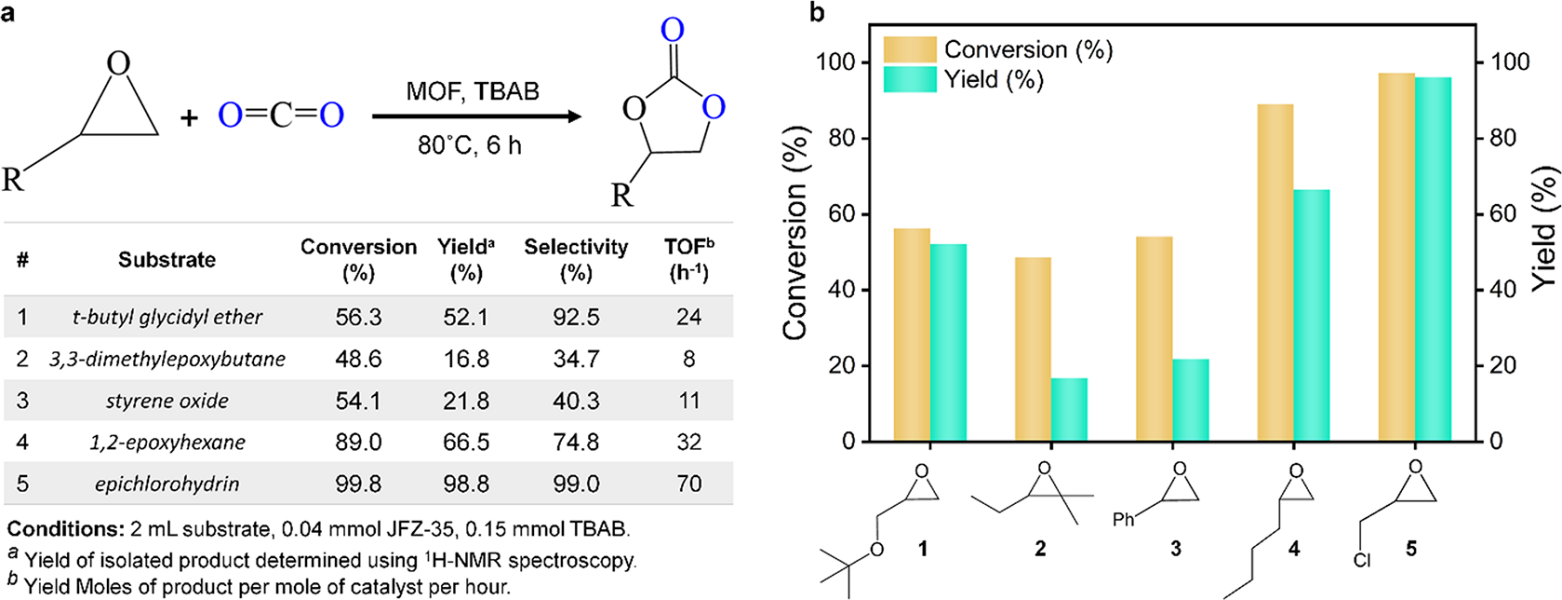
(a) Reaction scheme (above) and table (below) highlighting the results of catalytic cycloaddition reactions. (b) Corresponding plots showing the conversion and yields for different substrates using the UOW-1 catalyst.

Considering the favorable thermal stability (determined using TGA) and high Lewis acidity (discussed later) of UOW-1, its performance as a heterogeneous catalyst for CO_2_-mediated cycloaddition with epoxides was evaluated (Figure 4a). The experiments were initially optimized using 2-(chloromethyl)oxirane, commonly known as epichlorohydrin, as the model substrate (see the Experimental Section for details), and the products were analyzed using ^1^H nuclear magnetic resonance spectroscopy (^1^H NMR; Figure S5). The reactions were performed under solvent-free conditions in a pressurized round-bottom flask with a fixed amount of epichlorohydrin and a tetra-butyl ammonium bromide (TBAB) cocatalyst. The mass of the MOF catalyst, reaction temperature, and time were varied to identify the best working conditions. Consequently, 50 mg of UOW-1 (used without any heat treatment), 80 °C, and a short reaction time of 6 h were identified as the optimized conditions for the CO_2_ cycloaddition with the epichlorohydrin substrate achieving a conversion of ∼100%, a product (cyclic carbonate) yield of ∼99%, and a high turnover frequency (TOF) of 70 h^–1^ (Figure 4b, Table S4, and Figure S5). Decreased catalyst amounts of 20 and 10 mg showed slightly lower conversion rates and product yields of ∼94 and ∼90%, respectively (Figure S6a). Although longer reaction durations did not substantially change the reaction yields, lower temperatures decreased the catalytic activity (Figure S6b,c and Table S4). Control experiments performed by eliminating one component at a time did not lead to appreciable CO_2_ conversion (Figure S6d). In the absence of CO_2_, no product was formed, whereas with only the TBAB cocatalyst (without UOW-1), only 52% conversion was achieved. The high performance of the UOW-1 is further attested by the fact that in the absence of any cocatalyst, the UOW-1 catalyst still exhibited a conversion of >90% and a yield of ∼70% (Figure S6d). Interestingly, the dehydrated MOF shows only a trace amount of epoxide conversion, suggesting that the loosely bound guest water molecule, in the UOW-1 framework, might act as the nucleophile in the absence of TBAB. Recyclability tests for UOW-1 under the optimized conditions were carried out for the epichlorohydrin substrate for five consecutive cycles, which indicated the retention of performance and selectivity throughout all the cycles (Figure S7a). For better assessment on recyclability, the tests were further conducted at midway of the optimized reaction time for another five cycles (Figure S7b). The structural and chemical integrity of UOW-1 was well-preserved after the recyclability tests, as confirmed by PXRD and observed from the electron microscopy images (Figure S7c). Additionally, inductively coupled plasma-optical emission spectrometry (ICP-OES) of the postcatalysis sample revealed minimal leaching of yttrium (<0.1 ppm) into the liquid phase.

The UOW-1 catalyst was next applied for the cycloaddition of different types of epoxide substrates under the optimized conditions (50 mg of UOW-1, 80 °C, and 6 h). The broadening of the substrate scope helped to provide further insights and information on the effect of epoxide electrophilicity/nucleophilicity and steric factors on the CO_2_ cycloaddition reactions. As seen in Figure 4, the yields (determined using ^1^H NMR spectroscopy using 2,5-dimethylfuran as an internal standard; representative spectra shown in Figure S5) decreased with increasing steric hindrance and nucleophilicity at the carbon center of the epoxide substrates. The sterically bulky substrates find it difficult to diffuse into the MOF pores and interact with the catalytic centers, thereby having a lower propensity to react efficiently.^32^ The presence of an electron-donating “R” group, moreover, decreases the feasibility of Br^–^ attack during catalysis (see the mechanism in Figure S8).

**Figure 5:**
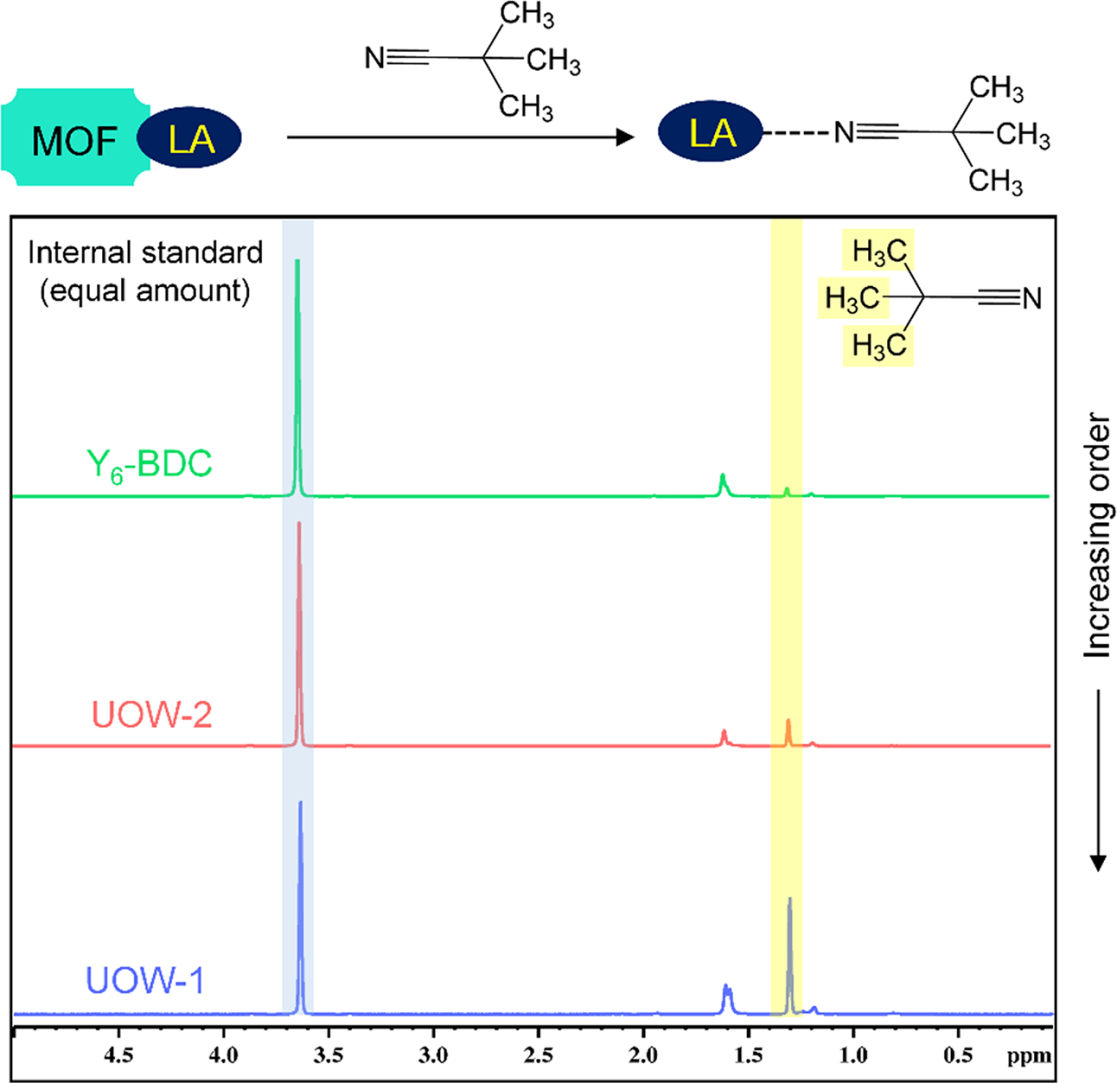
Lewis acidity (LA) determination using ^1^H NMR integrals of coordinated pivalonitrile in UOW-1 (bottom), UOW-2 (middle), and Y_6_-BDC (top).

The catalytic efficiency of UOW-1 toward CO_2_ cycloaddition using epichlorohydrin was further compared to UOW-2 and the BDC-based Y_6_-BDC MOF after heat activation (see the Experimental Section for details, Figure S9). Under identical conditions (50 mg of catalyst, 80 °C, and 6 h), the conversion and yields from UOW-2 and Y_6_-MOF were lower than that from UOW-1. The UOW-2 catalyst resulted in a conversion of ∼89% and a product yield of ∼79%. The Y_6_-BDC catalyst gave the poorest catalytic activity (conversion of ∼84%; product yield of ∼42%) among the three MOFs. From these results, we propose that the connectivity of surface atoms in the individual MOFs plays a major role in their catalytic activity. In the case of UOW-1, the yttrium centers are seven and eight coordinated, whereas UOW-2 and Y_6_-BDC have nine coordinated yttrium centers. The coordination unsaturation in UOW-1 likely contributes to its increased Lewis acidity, making it a better heterogeneous catalyst. This was further confirmed by quantifying and comparing the Lewis acidic sites present in the different MOFs using a Lewis base probe and ^1^H NMR analysis (Figure 5). We estimated the TOF based on the Lewis sites detected in UOW-1; although this assumes that the same sites are accessible to the substrate as the probe molecule, this gave a TOF value of ∼127.21.

The amount of Lewis acidic sites was found to be the highest for UOW-1 followed by UOW-2 and Y_6_-BDC,which is consistent with the catalysis trend for the different MOFs toward CO_2_ epoxidation.

### Kinetic and Mechanistic Analysis

**Figure 6:**
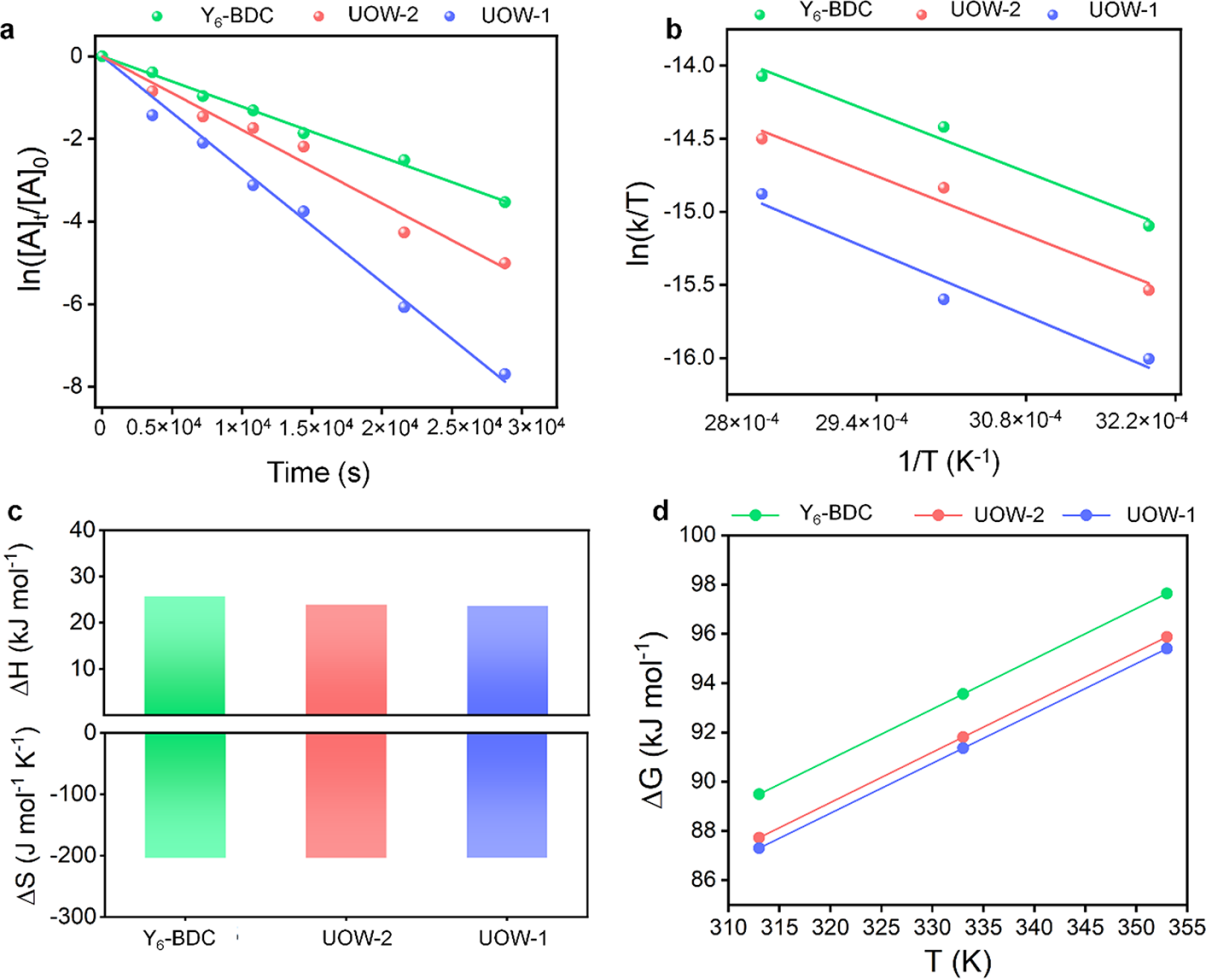
(a) Semilogarithmic plots of the epoxide concentration vs time (“[A]” indicates reactant concentration). (b) Eyring plots for the different catalysts. (c) Δ*H*^≠^, Δ*S*^≠^, and (d) Δ*G*^≠^ for the different systems determined using the Eyring plots. Conditions: 8 mL of epichlorohydrin, 0.16 mmol of UOW-1, and 0.6 mmol of TBAB, stirring.

The reaction kinetics for the different MOFs toward CO_2_ cycloaddition was further probed at 40, 60, and 80 °C. As shown in Figure 6a and Figure S10, semilogarithmic plots of the epichlorohydrin concentration “[A]” vs time indicate that the reaction follows first-order kinetics.

As expected, the reactions with UOW-1 show the highest rate constants of 0.9 × 10^–4^, 1.8 × 10^–4^, and 2.7 × 10^–4^ s^–1^ at 40, 60, and 80 °C, respectively (Table S5), which is consistent with its highest catalytic efficiency. The rate constants for reactions with UOW-2 and Y_6_-BDC are lower and shown in Tables S6 and S7.

The Eyring plots for the reactions (Figure 6b) were used to determine the enthalpy (Δ*H*^≠^) and entropy (Δ*S*^≠^) of activation from the reactants to the transition state (Figure 6c and Table S8). The Δ*H*^≠^ for the reactions decreases in the order Y_6_-BDC > UOW-2 > UOW-1, which is consistent with experimental observations. The lowest Δ*H*^≠^ for UOW-1 (23.6 kJ mol^–1^) suggests the lowest kinetic barrier, making the reaction most feasible. Additionally, the highest Δ*S*^≠^ for UOW-1 possibly helps to increase the rotational/conformational degrees of freedom associated with epoxide coordination.^33^ The Gibbs free energy (Δ*G*^≠^) for the different MOFs was also calculated (Table S9). As evident from Figure 6d, UOW-1 shows the lowest Δ*G*^≠^ followed by UOW-2 and finally Y_6_-BDC, which exhibits a significant jump in the Δ*G*^≠^, corroborating the experimental observations during catalysis.

The results suggest that the synergistic interaction between the metal center and the epoxide plays a vital role during catalysis, and therefore, a plausible mechanism for the cycloaddition reaction is proposed for the FDC-based MOFs in accordance with previous mechanistic insights (Figure S9).^34^ First, the Y^3+^ Lewis acidic center of the MOF coordinates with the epoxide O, thereby activating the ring for facile nucleophilic attack. The Br^–^ from TBAB then attacks the carbon resulting in epoxide ring opening, which is followed by CO_2_ insertion. Finally, intramolecular cyclization results in the formation of the corresponding cyclic carbonate with the concomitant regeneration of the catalyst.

We attempted to measure the surface area of UOW-1 using nitrogen adsorption but found the structure to be unstable to prolonged heat treatment under vacuum, despite its stability to heating in air (Figures S7d and S11). This supports the view that catalysis takes place at the surface of the MOF crystallites, and this is entirely reasonable, given that the bulky substrate molecules are highly unlikely able to diffuse into the structure, even if potential pore spaces were available.

### Comparison with Representative Systems

**Figure 7:**
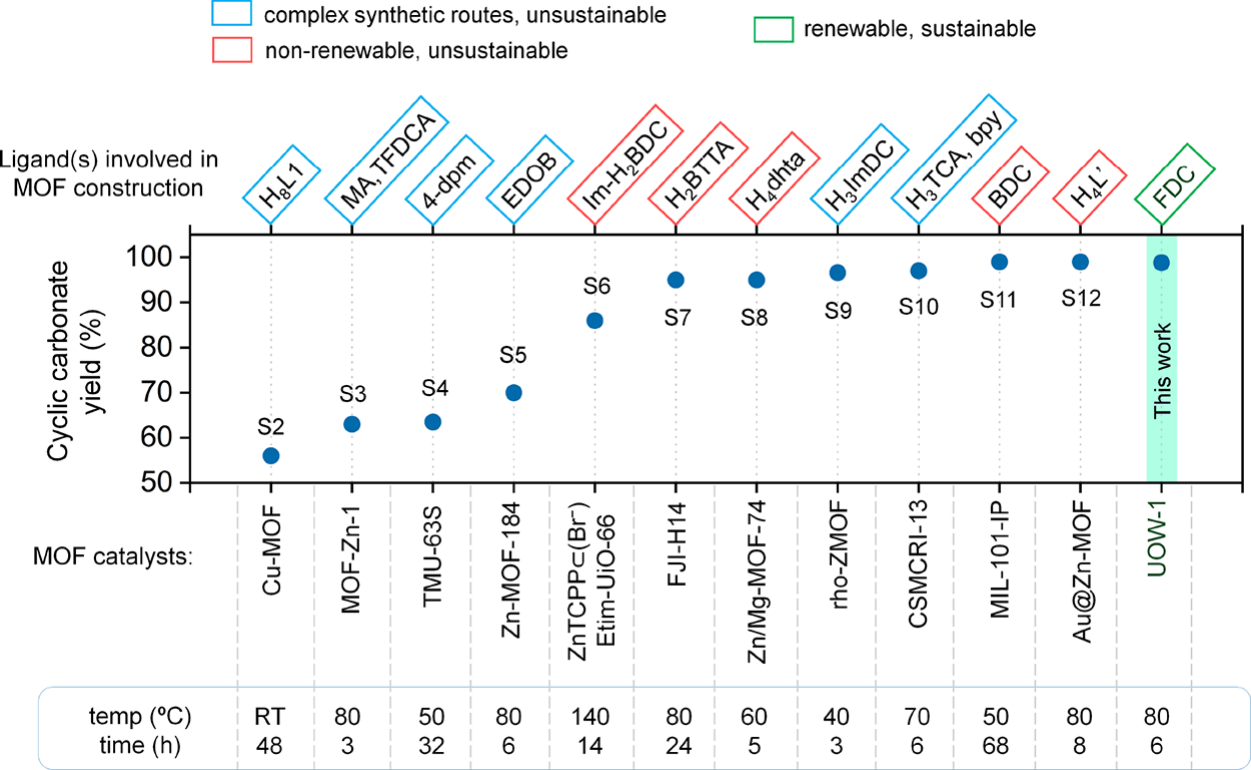
Comparison of our FDC-based UOW-1 MOF catalyst toward CO_2_ epoxidation using the epichlorohydrin model substrate with other representative MOF catalysts reported in the literature. For further details (including ligand abbreviations, catalyst and cocatalyst amounts, and corresponding references), see Table S10.

While a number of MOF-based catalysts have been explored for CO_2_ epoxidation reactions in recent times, a majority of such MOFs either consist of ligands derived from nonrenewable sources such as fossil fuels,^35,36^ or are constructed using complex linkers,^37,38^ which are synthetically challenging and therefore not scalable. Figure 7 shows a comparison of various MOF catalysts toward CO_2_ epoxidation with our best UOW-1 catalyst. It can be seen that the catalytic efficiency of UOW-1, which contains sustainable FDC linkers, is already comparable to the best existing candidates (with yields of >99%). Moreover, using UOW-1, the concentration of the cocatalyst required for carrying out the reaction is also minimal (Table S10). To the best of our knowledge, our work presents the first report of the FDC-based MOF catalyst for CO_2_ utilization. These results pave the way for new directions in the domain of MOF-driven catalysis, where a range of different MOFs with green and sustainable linkers may be designed and applied for various catalytic processes in the future.

## Conclusions

We have synthesized and characterized a novel FDC-based MOF (UOW-1) with a unique topology. Benefiting from Lewis acidic sites, the MOF holds promise as an efficient recyclable catalyst for the heterogeneous cycloaddition of CO_2_ to epichlorohydrin under solvent-free conditions yielding cyclic carbonates. With a minimal cocatalyst requirement and only 4 wt % UOW-1, a promising catalytic activity (∼100% conversion, ∼99% selectivity, and 70 h^–1^ TOF) toward solvent-free CO_2_ cycloaddition was achieved, which is either better than or comparable to most processes employing MOF catalysts with complex or nonrenewable linkers. Insights into the overall catalytic process were obtained through diversifying the substrate scope and kinetic analysis. Our results open exciting new avenues for the exploration of green, FDC-based MOFs toward CO_2_ fixation.

## Experimental Section

### Materials

Yttrium chloride hexahydrate (Sigma-Aldrich, 99.99%), 2,5-furandicarboxylic acid (Sigma-Aldrich, 97%), sodium hydroxide (Fisher Scientific, 98%), terephthalic acid (Sigma-Aldrich, 97%), epichlorohydrin (Thermo Scientific, 99%), 1,2-epoxyhexane (Acros, 97%), styrene oxide (Thermo Scientific, 97%), 3,3-dimethylepoxybutane (ABCR), *t*-butyl glycidyl ether (ABCR), tetra-butyl ammonium bromide (Sigma-Aldrich), dry ice, and 2,5-dimethylfuran (Thermo Scientific, 99%) were used. Solvents methanol, deionized water, isopropanol, acetonitrile, dimethyl sulfoxide, and deuterated chloroform were used. All reagents were commercially available and purchased in high purity. These were used without further purification.

### Synthesis of UOW-1

H_2_FDC (0.3 mmol, 46.8 mg) and NaOH (0.3 mmol, 12.0 mg) were added to 2 mL of CH_3_OH, and the mixture was stirred for 20 min to obtain a white suspension. YCl_3_·6H_2_O (0.1 mmol, 30.3 mg) was then added into the suspension and stirred. After adding 1 mL of H_2_O into the reaction mixture, the suspension turned into a colorless solution. The resulting solution was sealed in a Teflon-lined autoclave and heated at 110 °C for 48 h. After slow cooling to room temperature (5 °C h^–1^), colorless crystals of UOW-1 were obtained.

### Synthesis of UOW-2

YCl_3_·6H_2_O (0.2 mmol, 60.6 mg), H_2_FDC (0.4 mmol, 62.4 mg), and NaOH (0.4 mmol, 16.0 mg) were added to a solution of 2 mL of H_2_O, 4 mL of CH_3_CN, and 0.2 mL of DMSO. Caution: DMSO is known to be highly reactive when heated in mixed solvents,^39^ although in our experiments, no such issues were observed. The mixture was stirred to obtain a white suspension. The suspension was sealed in a Teflon-lined autoclave and heated at 110 °C for 48 h. After slow cooling to room temperature (3 °C h^–1^), colorless crystals of UOW-2 were obtained.

### Synthesis of Y_6_(BDC)_7_(OH)_4_(H_2_O)_4_ (Y_6_-BDC)

YCl_3_·6H_2_O (1.81 g, 6 mmol), Na_2_BDC (1.575 g, 7.5 mmol), NaOH (2.2 g, 2 M), and H_2_O (50 mL) were added to a 100 mL Teflon-lined autoclave. The autoclave was then sealed and heated to 190 °C for 72 h. After cooling, the product was obtained by vacuum filtration and washed 3× with deionized water followed by 3× with isopropanol. The product was dried at 70 °C overnight. Before use, the MOF was activated to remove terminally bound water molecules. Activation was carried out by heating the material at 200 °C for 2 h.

### Materials Characterization and Instrumentation

Powder X-ray diffraction (PXRD) patterns of different samples were recorded using a Siemens D5000 diffractometer equipment with Cu Kα_1/2_ radiation with data being recorded in the Bragg–Brentano mode with a step size of Δ2θ = 0.02° and at a 4 s per step. The morphology of the catalyst was characterized by a Zeiss Supra 55-VP field emission scanning electron microscope (FESEM). The thermogravimetric (TGA) analysis was performed using a Mettler Toledo TGA/DSC1 instrument under ambient air pressure and a heating rate of 10 °C min^–1^. The samples were heated in air from 25 to 1000 °C. The ^1^H NMR analysis was carried out using a Bruker Avance III HD 300 MHz instrument. Inductively coupled plasma (ICP) spectroscopy was performed by a Varian Vista MPX ICP-OES system by Medac Limited for the chemical analysis of the catalyst.

#### Single-Crystal XRD

Single-crystal XRD was carried out by mounting suitable crystals on glass fibers with silicon grease and placing them on a Rigaku Oxford Diffraction Supernova diffractometer with a dual source (Cu at zero) equipped with an AtlasS2 CCD area detector. The crystals were kept at 293(2) K during data collection. The structures were solved using the Olex2 package with the ShelXT5 structure solution program using intrinsic phasing and refined with the ShelXL6 refinement package using least squares minimization. The topology was calculated by TOPOS*.* All the crystallographic and structure refinement data of the UOW-1 and UOW-2 MOFs are summarized in Tables S1 and S3.

### Catalytic Nonredox CO_2_ Cycloaddition Reactions

In a typical reaction, 2 mL of epichlorohydrin (25.5 mol), 50 mg of the MOF catalyst, and 50 mg of TBAB were added together in a three-neck reaction flask equipped with a stirrer bar. To this mixture, 5 g of dry ice was added, and thereafter, the reaction flask was sealed with a rubber balloon. The flask was then placed in a preheated oil bath and stirred for the requisite time. Once the reaction was completed, the mixture was allowed to cool down to room temperature. Thereafter, the catalyst was separated through filtration, and the product was analyzed via ^1^H NMR spectroscopy using 2,5-dimethylfuran as the internal standard and deuterated chloroform as the solvent. A similar procedure was followed for reactions with different epoxide substrates.

### Recyclability Tests

For the recyclability tests, the recovered catalyst was washed 3 times with isopropanol and dried in a fan oven. Thereafter, the oven-dried catalyst was further used for CO_2_ cycloaddition using the abovementioned protocol. This process was followed for five consecutive cycles. NMR spectroscopy revealed high selectivity of the catalyst, whereas for all studied epoxides, it was observed that the cyclic carbonate was the main product with an absence of polycarbonate or hydrolysis products.

### Quantitative ^1^H NMR Spectroscopy

Samples were spiked with a known quantity of the internal standard (2,5-dimethylfuran). The amount of the analyte in the sample was calculated using eq 1:
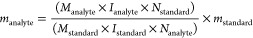
1where *I*_analyte_ is the integral of the analyte peak, *N*_analyte_ is the number of protons corresponding to the analyte peak, *M*_analyte_ is the molar mass of the analyte, and *m*_standard_ is the known mass of the standard in the sample.

### Lewis Acidity Characterization and Quantification

To quantify accessible Lewis acid sites in the MOFs, each of them (UOW-1, UOW-2, and Y_6_-BDC) was treated with 20 equiv of trimethyl acetonitrile as a steric bulky Lewis base probe for 4 h.^40^ Prior to the Lewis acidity measurement (and also for catalysis), the crystals were ground. The resultant solid was centrifuged and washed with toluene thoroughly to remove excess uncoordinated base probe. The resultant material was dried and digested in D_3_PO_4_/DMSO-*d*_6_. The resultant mixture was then analyzed by ^1^H NMR. A 75 mg amount of 1,4-dioxane was used as the internal standard to quantify the amount of coordinated trimethyl acetonitrile in the case of each system. The coordinated trimethyl acetonitrile was found to be the highest for UOW-1 followed by UOW-2 and Y_6_-BDC.

### Kinetic and Thermodynamic Analysis

The kinetic analysis of the cycloaddition reaction (with different catalysts) was performed by determining the concentration of the epichlorohydrin substrate/reactant (denoted as [A]_*t*_, where *t* is the time) at different time intervals during the reaction. The ln([A]_*t*_/[A]_0_) vs the time plot was then fitted using first-order kinetics according to eq 2 in order to determine the rate constant (*k*) of the reaction. The goodness-of-fit (*R*^2^) for the linear fit was ∼1, which suggests that the reaction indeed follows pseudo-first-order kinetics.
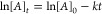
2

The rate constant for the reactions at different temperatures (40, 60, and 80 °C) was further used to determine the thermodynamic parameters (i.e., the enthalpy (Δ*H*^≠^) and entropy (Δ*S*^≠^) of activation from the reactants to the transition state) according to the Eyring equation (eq 2) shown below, where “*k*” is the rate constant, *T* is the temperature, *R* is the universal gas constant (8.314 J K^–1^ mol^–1^), *k*_B_ is the Boltzmann constant (1.38 × 10^–23^ m^2^ kg s^–2^ K^–1^), and *h* is the Planck constant (6.626 × 10^–34^ m^2^ kg s^–1^).
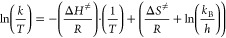
3

The Δ*H*^≠^ and Δ*S*^≠^ determined from the slope and the intercept of the ln(*k*/*T*) vs (1/*T*) plot, respectively, were further used to estimate the Gibbs free energy of the reaction with different MOF catalysts according to eq 4:
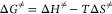
4

## Notes

CCDC 2165364 and 2165365 contain the supplementary crystallographic data for UOW-2 and UOW-1, respectively: these data can be obtained free of charge from The Cambridge Crystallographic Data Centre *via*www.ccdc.cam.ac.uk/structures.

## Supporting Information Available

Description of crystal structures of UOW-1 and UOW-2; thermogravimetric analysis of UOW-1 and UOW-2; numerical data from CO_2_ cycloaddition reactions; solution NMR spectra; further catalytic results and recyclability data; proposed mechanism; powder XRD of stability of UOW-1 (PDF)
