# Supplementary material for: Nonredox CO2 Fixation in Solvent-Free Conditions Using a Lewis Acid Metal–Organic Framework Constructed from a Sustainably Sourced Ligand
Source: Inorg Chem. 2022 Nov 10;61(46):18536–44. doi: 10.1021/acs.inorgchem.2c02749 (PMC9682481; doi:10.1021/acs.inorgchem.2c02749)
Supplement: Supplementary file 1 — ic2c02749_si_001.pdf [file ic2c02749_si_001.pdf]

Supporting Information

Non-redox CO_2-fixation in Solvent-free Conditions Using a Lewis Acid MOF Constructed from a Sustainably Sourced Ligand

Satarupa Das,[1] Jinfang Zhang,[1,2] Thomas W. Chamberlain,[1] Guy J. Clarkson,[1] Richard I. Walton[1]*

1. Department of Chemistry, University of Warwick, Coventry, CV4 7AL, UK.
2. International Joint Research Center for Photoresponsive Molecules and Materials, School of Chemical and Material Engineering, Jiangnan University, Wuxi 214122, P. R. China
E-mail: *R.I.Walton@warwick.ac.uk*

Structural analysis of UOW-1

In case of UOW-1, Y1 centers are coordinated by seven O atoms (from one H_2O, one L1, two L2, one L3, one L4 and one L5) to present a distorted pentagonal bipyramid geometry (FigureS1,S2). Y2 and Y3 are eight-coordinated and exhibit distorted dodecahedral geometries with different coordination environments (Figure S2). Y2 centers are coordinated by eight O atoms (from four H_2O, one L1, two L3 and one L4), whereas Y3 centers are surrounded by one H_2O, one L2, one L3 two L4 and two L5 (Figure S1). The Y−O bond-lengths are in the range of 2.262 (2) −2.6926 (18) Å, and the O−Y−O bond angles vary from 51.00 (6) °−171.22 (7)°. Further structural analysis revealed that L1 connects with one Y1 and one Y2 in a μ_2-$\eta^1\eta^1$ coordination fashion. L2 links to two Y1 and one Y3 to exhibit a μ_3-$\eta^1\eta^2\eta^1$ coordination fashion. L3 and L4 show the same μ_4-$\eta^1\eta^1\eta^1\eta^1$ coordination fashion. While L3 bonds to one Y1, two Y2 and one Y3, L4 connects with one Y1, one Y2 and two Y3. L5 displays μ_3-$\eta^1\eta^1\eta^1$ coordination via linking to one Y1 and two Y3.

In this 3D-framework, Y1 and Y3 units exhibit 6-connected modes: each Y1 links to one Y2 by L1 bridge, two L2, one L3, one L4 and one L5; Y3 centers are connected by one L2, one L3, two L4 and two L5 units. Y2 connects with one Y1 by L1 bridge, two L3 and one L4 units to form 4-connected mode. L2 links to two Y1 and one Y3, and L5 bonds to one Y1 and two Y3; therefore, they exhibit 3-connected modes. L3 and L4 are in 4-connected modes, where L3 bonds to one Y1, two Y2 and one Y3; L4 connects with one Y1, one Y2 and two Y3. Topologically, L1 is treated as linker; Y1 and Y3 centers can be regarded as 6-connected nodes; L2 and L5 act as 3-connected nodes; Y2, L3 and L4 are 4-connected nodes. The Schläfli symbols are $(3{\cdot}4^3{\cdot}6^2{\cdot}7^5{\cdot}8^4)$ for Y1, $(3{\cdot}4^2{\cdot}5{\cdot}6{\cdot}7)$ for Y2 and L3, $(4^5{\cdot}6^7{\cdot}8^3)$ for Y3, $(4{\cdot}6^2)$ for L2, $(4^3{\cdot}6^3)$ for L4, and $(4{\cdot}6{\cdot}8)$ for L5. Hence, UOW-1 can be regarded as a (3,3,4,4,4,6,6)-c topology with a point symbol of $(4{\cdot}6^2)$ $(4{\cdot}6{\cdot}8)$ $(3{\cdot}4^2{\cdot}5{\cdot}6{\cdot}7)_2(4^3{\cdot}6^3)$ $(3{\cdot}4^3{\cdot}6^2{\cdot}7^5{\cdot}8^4)$ $(4^5{\cdot}6^7{\cdot}8^3)$.

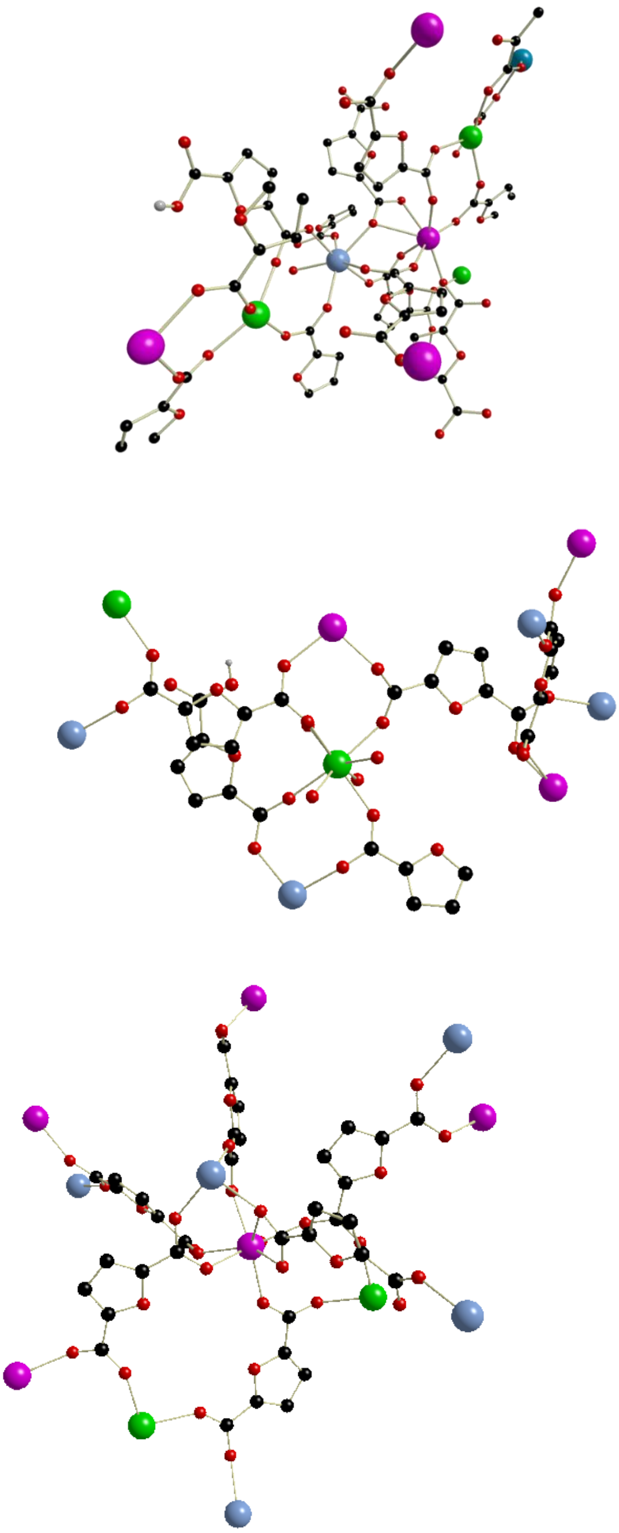

Figure S1: coordination and connectivity of different yttrium centers of UOW-1. Colour codes: Y1 - blue, Y2 - green, Y3 - pink, C - black, O - red, H – grey.

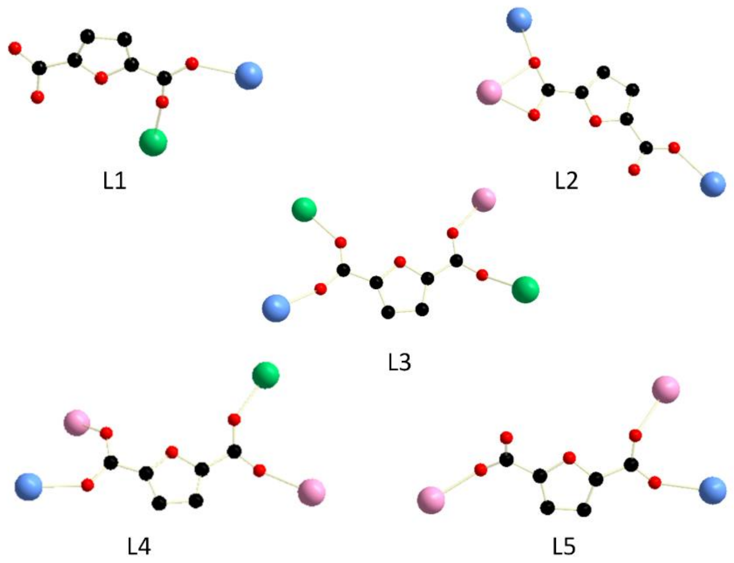

`

Figure S2: different coordination mode of L in JFZ-35. L refers to furan 2,5-dicarboxylic acid. Colour codes: Y1 - blue, Y2 - green, Y3 - pink, C - black, O - red, H – grey.

Table S1. Crystal data and refinement conditions for UOW-1.

**Structure Code**	**UOW-1**
**Empirical formula**	*$C_{31.75}H_{30.86}O_{34.25}Y_3$*
**Formula weight**	*1227.16*
**Radiation**	*CuKα (λ=1.54184)*
**Temperature**	*100(2) K*
**Crystal system**	*monoclinic*
**space group**	*P 21/c*
**Crystal density**	*1.866*
**a /Å**	*18.29270(10)*
**b /Å**	*10.07500(10)*
**c /Å**	*24.88350(10)*
**α /°**	*90*
**β /°**	*107.7720(10)*
**γ /°**	*90*
**Volume /Å^3**	*4367.15(6)*
**Z**	*4*
**2q range for data collection/°**	*3.7210 to 79.7270*
**Absorption coefficient**	*6.245*
**Completeness to theta**	*99.75*
**Max. and min. transmission**	*0.635 and 0.368*
**Data/restraints/parameters**	*9394/0/646*
**Absorption correction**	*spherical harmonics - Frame scaling*

**F(000)**	*2449*
**Crystal size /mm^3**	*0.2 ×0.12× 0.08*
**Index ranges**	*-23 ≤ h ≤ 23, -11 ≤ k ≤ 12, - 30≤ l ≤ 31*
**Reflections collected**	*43732*
**Goodness-of-fit on F2**	*1.076*
**Independent reflections**	*9394(R_{int}= 0.061)*
**Final R indexes [I>=2σ (I)]**	*R1= 0.0311, WR2= 0.0858*
**Final R indexes [all data]**	*R1= 0.0327, WR2= 0.0869*
**Largest diff. peak/hole / e Å^{-3}**	*1.407/ -1.053*

Thermogravimetric Analysis of UOW-1.

The thermal stability and chemical confirmation of the MOF was investigated by thermogravimetric analysis (TGA). TGA of the as-synthesised UOW-1 revealed gradual mass loss up to about 400ºC triggered by the removal of solvent molecules, coordinated H_2O and FDC ligands, followed by the thermal decomposition of the framework to yield Y_2O_3.

Table S2. Assignment of thermogravimetric analysis of UOW-1

Temp range (˚C)	Theoretical mass loss (%)	Experimental mass loss (%)	Assignment	Chemical Change
84-87	95.5	96.8	removal of guest water molecules	$[\{Y_3(HFDC)(FDC)_4(H_2O)_6\}\cdot 3H_2O]_n$ → $[\{Y_3(HFDC)(FDC)_4(H_2O)_6\}]_n$
175-185	86.5	86.7	Removal of host water molecules	$[\{Y_3(HFDC)(FDC)_4(H_2O)_6\}\cdot 3H_2O]_n$ → $[\{Y_3(HFDC)(FDC)_4\}]_n$
388-400	73.6	77.4	removal of partial FDC ligands	$[\{Y_3(HFDC)(FDC)_4(H_2O)_6\}\cdot 3H_2O]_n$ → $[\{Y_3(HFDC)(FDC)_3\}]_n$
535-555	28.8	30.4	Combustion generating Y_2O_3	$[\{Y_3(HFDC)(FDC)_4(H_2O)_6\}\cdot 3H_2O]_n$ → Y_2O_3

Structural Analysis of UOW-2

UOW-2 crystallizes in the triclinic crystal system with space group of *P-1* and is isostructural to the reported MOFs $\{[Ln_2(FDA)_2(H_2O)_{10}]\cdot FDA\cdot 6H_2O\}_n$ (Ln = Dy, Eu, Gd).[1] The asymmetric unit of UOW-2 contains two Y^{3+} (Y1 and Y2), two fully deprotonated FDC^{2-} ligands, ten coordinated H_2O molecules, one free FDC^{2-} anion, and six free H_2O molecules (Figure S3). Y1 and Y2 have the same coordination environment (FigureS3). Each Y is nine-coordinated by four O atoms from two FDC^{2-} ligands and five O atoms from five coordination H_2O molecules to form a distorted monocapped square antiprism. The bond lengths of Y−O are in the range of 2.324 (2) −2.550 (2) Å, and bond angles of O−Y−O vary from 52.50 (8) to 147.88 (10)°. Two crystallographic independent FDC^{2-} ligands show the same μ_2-$\eta^1\eta^1\eta^1\eta^1$ coordination fashion. They act as double-chelated bridges, and link with neighboring Y to generate a 1D wave-like chain.

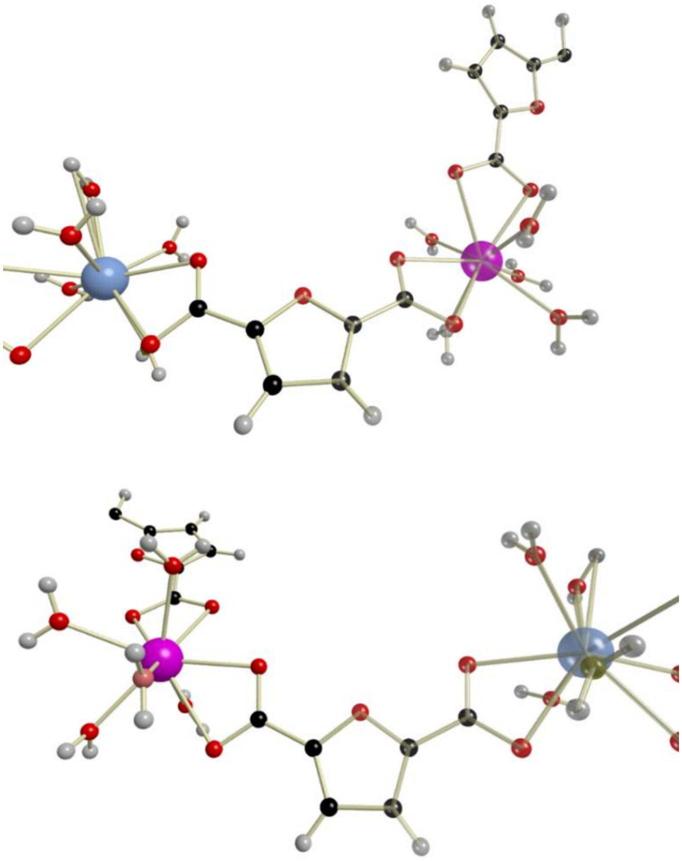

Figure S3: coordination environment of different yttrium centers of UOW-2. Colour codes: Y1 - blue, Y2 - pink, C - black, O - red, H – grey

Table S3. Crystal data and refinement conditions for UOW-2 MOF.

Structure Code	UOW-2
Empirical formula	$C_{18} H_{36} O_{31} Y_2$
Formula weight	926.29
Radiation	MoKα (λ=0.71073)
Temperature	150(2) K
Crystal system	triclinic
space group	$P\bar{1}$
Crystal density	1.787
a /Å	10.7142(4)
b /Å	10.8467(5)
c /Å	15.0087(7)
α /°	83.624(4)
β /°	85.560(4)
γ /°	84.324(4)
Volume /Å^3	1721.02(14)
Z	2
2q range for data collection/°	2.737 to 30.923
Absorption coefficient	3.457
Completeness to theta	99.65
Max. and min. transmission	1 and 0.74563
Data/restraints/parameters	9594/72/550
Absorption correction	spherical harmonics - Frame scaling
F(000)	940
Crystal size /mm^3	0.3 ×0.18× 0.01
Index ranges	$-15 \leq h \leq 15$, $-15 \leq k \leq 15$, $-21 \leq l \leq 21$
Reflections collected	52993
Goodness-of-fit on F2	1.04
Independent reflections	6511(R_{int}= 0.061)
Final R indexes [I>=2σ (I)]	R1= 0.061, WR2= 0.0806
Final R indexes [all data]	R1= 0.1082, WR2= 0.0924
Largest diff. peak/hole / e Å^{-3}	0.957/ -0.726

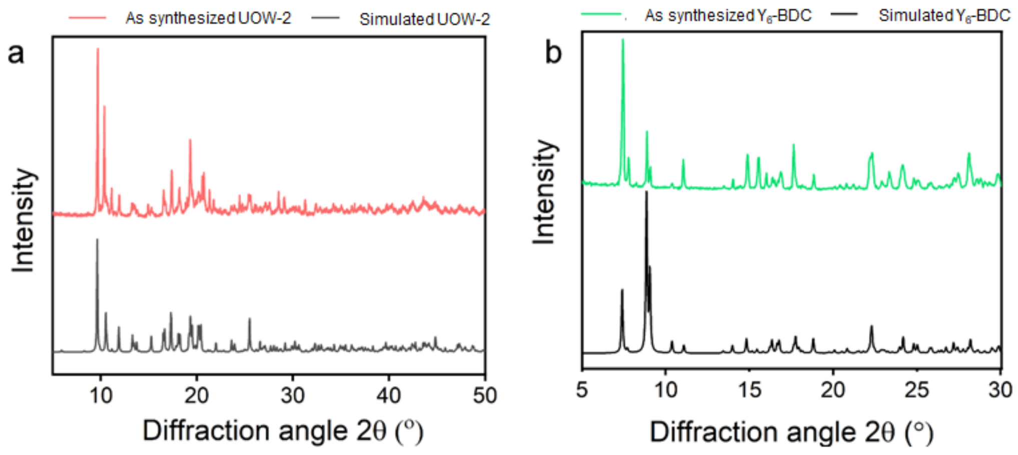

Figure. S4. Powder X-ray diffraction pattern of UOW-2 and Y_6-BDC MOF plotted with the simulated pattern.

Table S4. Numerical data: Conversion, yields, selectivities of the different CO_2 cycloaddition reactions with epichlorohydrin substrates using the different MOF systems (bold indicates the optimised conditions).

**Sl. No.**	**MOF**	**Catalyst Amount (mg)**	**Reaction Time (h)**	**Temp. (°C)**	**Conversion (%)**	**Yield (%)**	**Selectivity (%)**
1	UOW-1	20	24	20	45	12.7	28.2
2	UOW-1	20	24	40	70	25	35.7
3	UOW-1	20	24	60	94.4	69.7	73.8
4	UOW-1	20	24	80	100	96.8	96.8
5	UOW-1	20	18	80	100	95.6	95.6
6	UOW-1	20	12	80	99	92.4	93.3
7	UOW-1	20	6	80	93.6	89.7	95.8
8	UOW-1	10	6	80	89.9	72.2	80.3
9	UOW-1	**50**	**6**	**80**	99.8	98.8	98.9
10	UOW-2	**50**	**6**	**80**	88.9	78.5	88.3
11	Y_6-BDC	**50**	**6**	**80**	83.9	41.7	49.7
12	YCl_3 salt	**50**	**6**	**80**	69	43.6	63.2
**Controls with the best UOW-1 catalyst (under optimised conditions)**							
13		*No CO_2*			1	0	
14		*No TBAB*			92.1	69.3	75.2
15		*No MOF*			52.3	41.6	79.5

Table S5. Data obtained from Eyring analysis, linear fits, and calculated rate coefficients for the cycloaddition of CO_2 and epichlorohydrin using UOW-1 catalyst at different temperatures.

Entry	T (K)	R^2	k (× 10^{-4} s^{-1})	ln (k/T)
1	313	0.9992	0.87±0.04	-15.09
2	333	0.9958	1.82±0.08	-14.42
3	353	0.9974	2.73 ± 0.11	-14.07

Table S6. Data obtained from Eyring analysis, linear fits, and calculated rate coefficients for the cycloaddition of CO_2 and epichlorohydrin using UOW-2 catalyst at different temperatures.

Entry	T (K)	R^2	k (× 10^{-4} s^{-1})	ln (k/T)
1	313	0.9973	0.56 (±0.04)	-15.54
2	333	0.9961	1.20 (±0.03)	-14.84
3	353	0.9918	1.78 (±0.09)	-14.50

Table S7. Data obtained from Eyring analysis, linear fits, and calculated rate coefficients for the cycloaddition of CO_2 and epichlorohydrin using Y_6-BDC catalyst at different temperatures.

Entry	T (K)	R^2	k (× 10^{-4} s^{-1})	ln (k/T)
1	313	0.9992	0.35 (±0.03)	-16.01
2	333	0.9961	0.57 (±0.02)	-15.59
3	353	0.9918	1.22 (±0.09)	-14.88

Table S8. Transition state thermodynamic parameters: $\Delta H^{\neq}$ and $\Delta S^{\neq}$, determined using Eyring plots.

Entry	Catalyst	y = mx + c	R^2	$\Delta H^{\neq}$ (kJ mol^{-1})	$\Delta S^{\neq}$ (J mol^{-1} K^{-1})
1	UOW-1	2841.4x+5.98	0.978	23.60 (±4.81)	-203.50 (±11.65)
2	UOW-2	2878.8x+6.29	0.972	23.93 (±3.36)	-203.83 (±16.27)
3	Y_6-BDC	3095.9x+6.17	0.970	25.73 (±1.89)	-203.72 (±9.81)

Table S9. Gibbs free energy of the transition state determined for three catalysts.

Entry	System	Temperature (K)	$\Delta G^{\neq}$ (kJ mol^{-1})
1	UOW-1	313	87.30 (±1.10)
		333	91.36 (±1.23)
		353	95.40 (±2.08)
2	UOW-2	313	87.73 (±0.98)
		333	91.81 (±1.31)
		353	95.88 (±1.36)
3	Y_6-BDC	313	89.49 (±1.55)

	333	93.56 (±2.22)
	353	97.64 (±2.82)

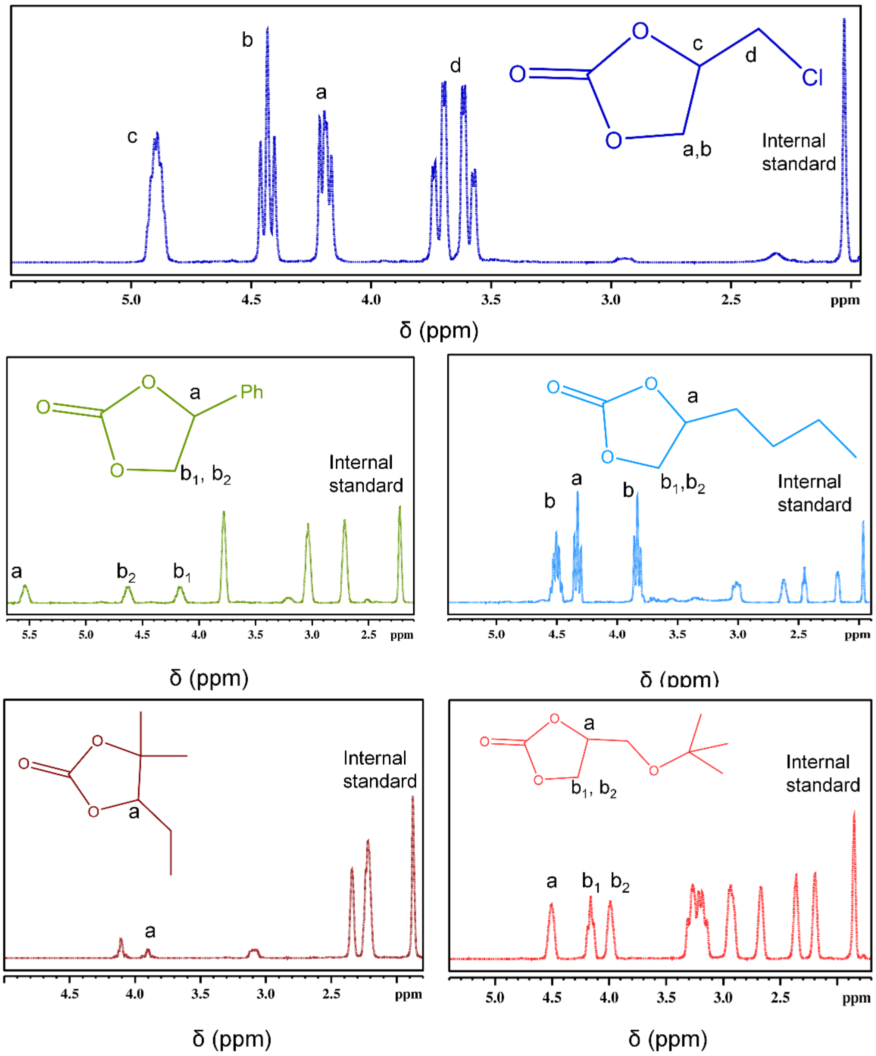

Figure S5. Representative ^{1}H NMR ($CDCl_3$, 300 MHz) spectra after the cycloaddition reactions of various substrates (from top to bottom: epichlorohydrin, 1,2-epoxyhexane, styrene oxide ,3,3-dimethylepoxybutane, t-butyl glycidyl ether) with CO_2 at 80 °C, 6 h using UOW-1.Internal standard- 2,5 dimethyl furan.

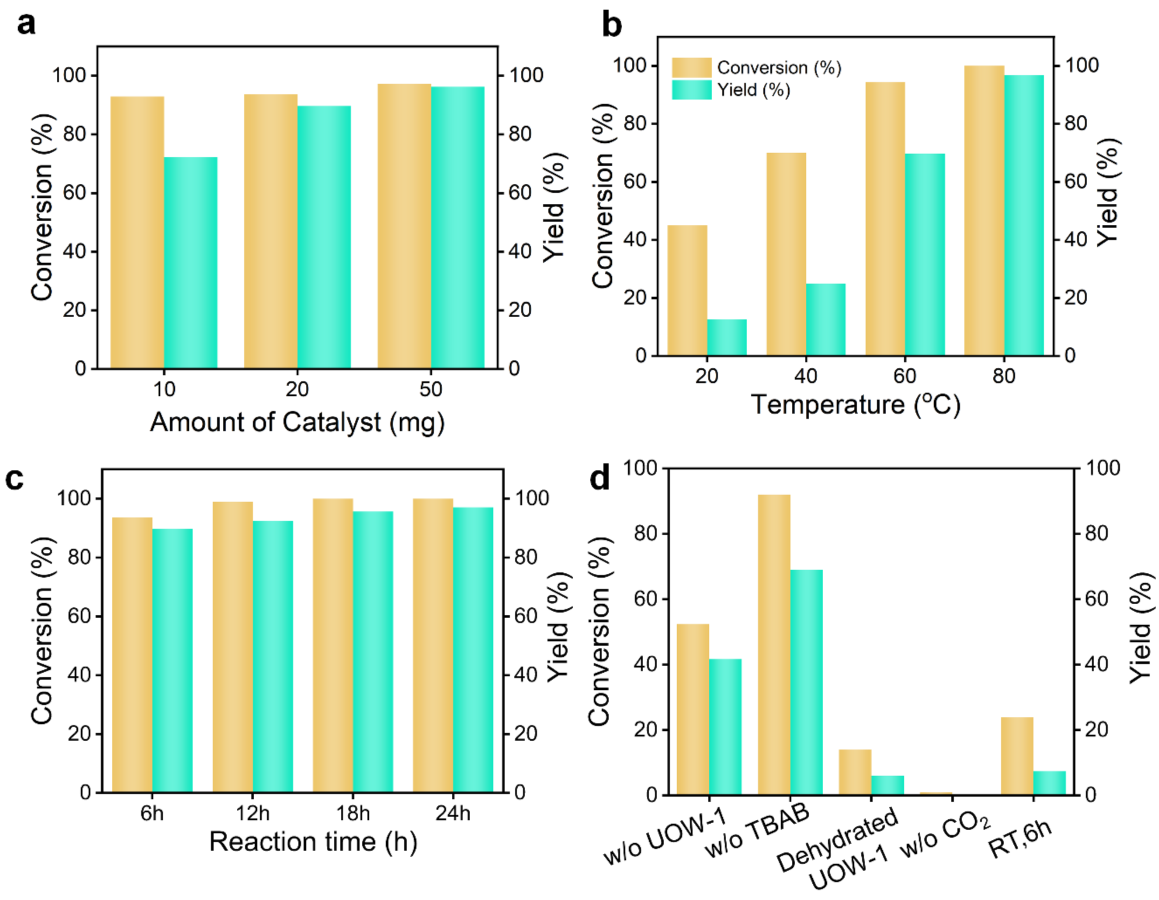

Figure S6. (a) Influence of catalyst amount on the cycloaddition of epichlorohydrin and CO_2 under optimised conditions: 80 °C, 6 h (b) Effect of reaction temperature and (c) reaction time on the cycloaddition of epichlorohydrin and CO_2 using UOW-1 catalyst, optimised conditions: 80°C, 20 mg catalyst (d) Control experiments performed by eliminating one parameter at a time using epichlorohydrin substrate and UOW-1 catalyst.

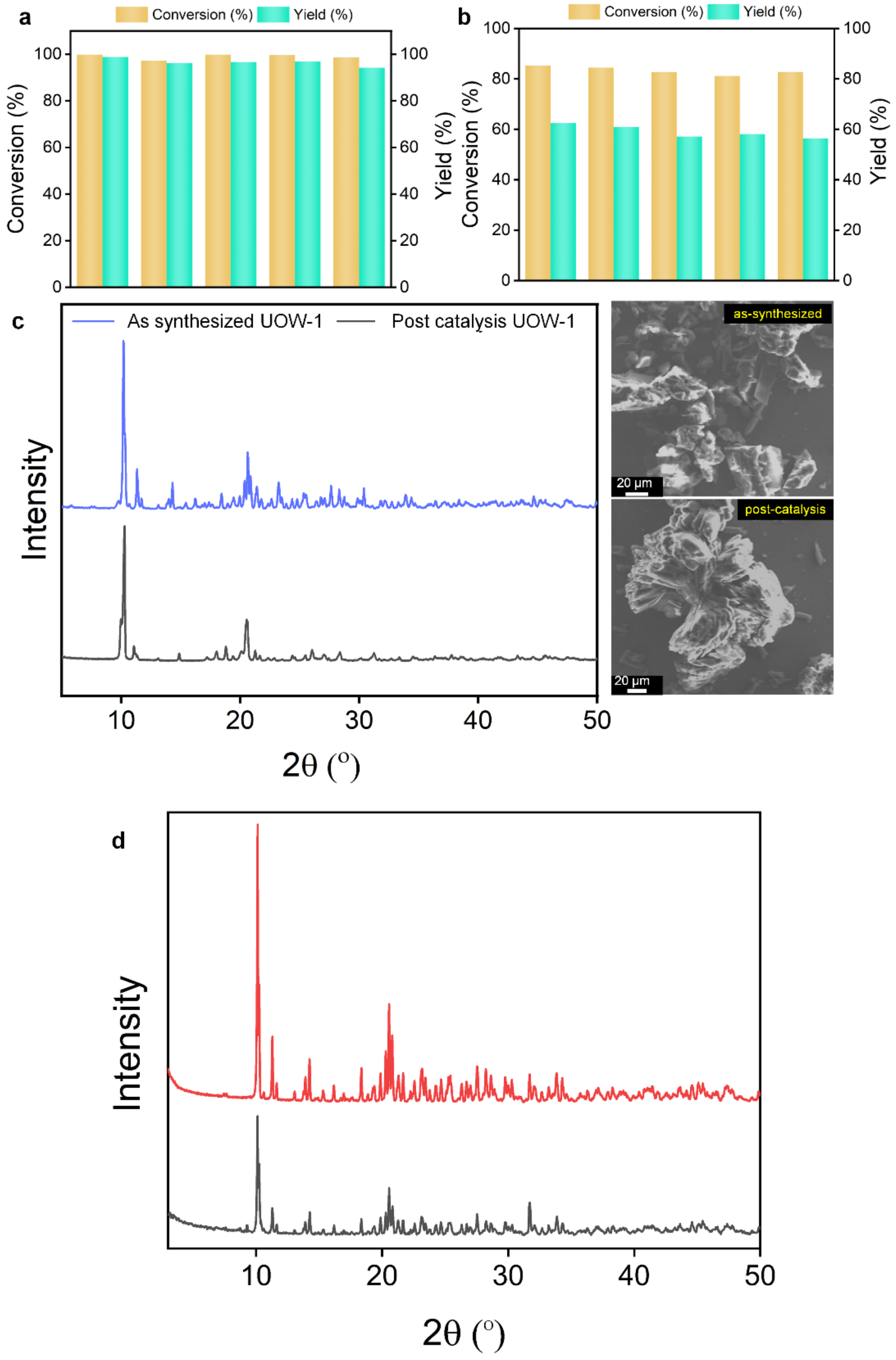

Figure S7. (a) Recyclability plots of UOW-1 with epichlorohydrin substrate under optimised conditions: 80°C, 6 h. (b) Recyclability plots of UOW-1 with epichlorohydrin substrate under optimised conditions: 80°C, 3 h. (c) PXRD pattern and SEM of as synthesised catalyst compared with post recyclability catalyst under optimised conditions: 80°C, 6 h. (d) Powder XRD after thermal activation (120 °C, 6 hours) and re-exposure to air, demonstrating stability (as synthesised, red, after heat treatment, black) .

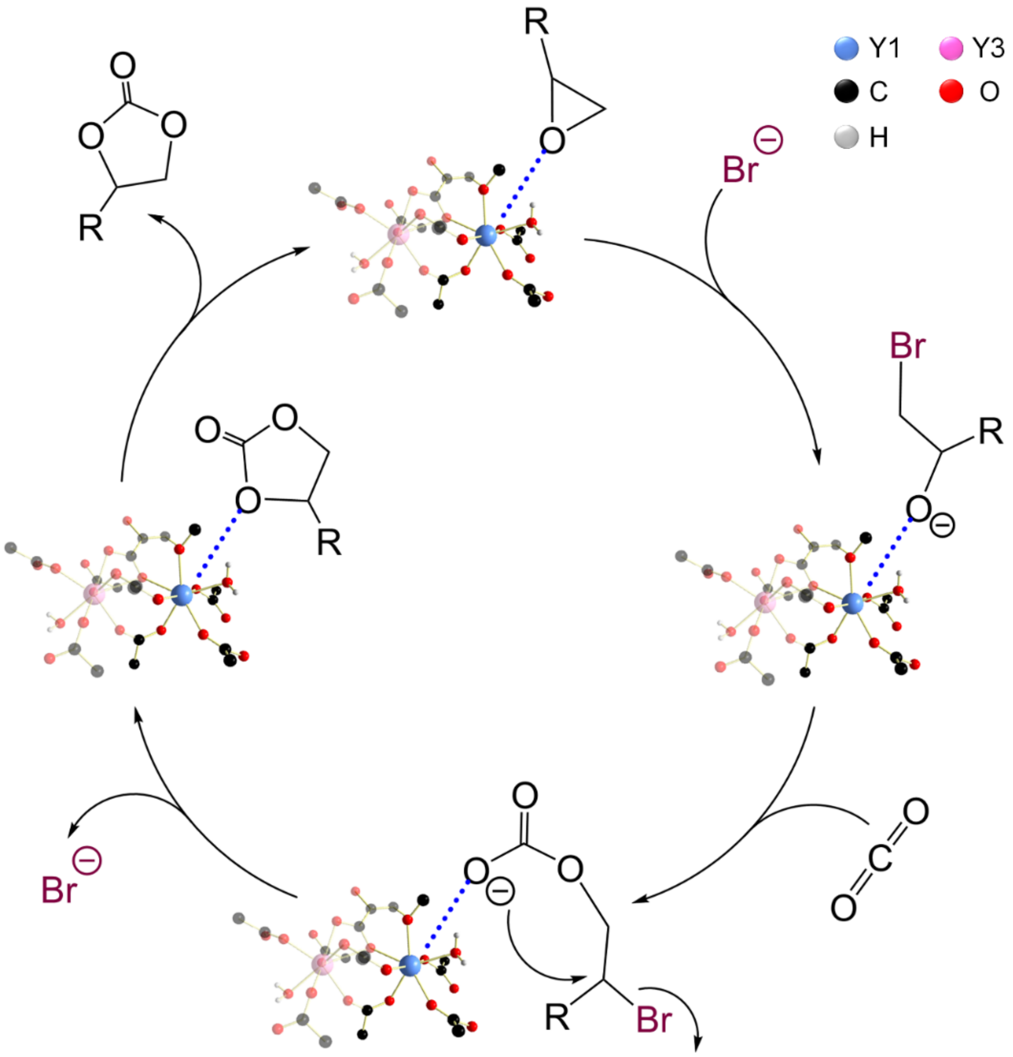

Figure S8. Proposed mechanism for the cycloaddition of CO_2 and epoxide in presence of UOW-1 MOF and TBAB.

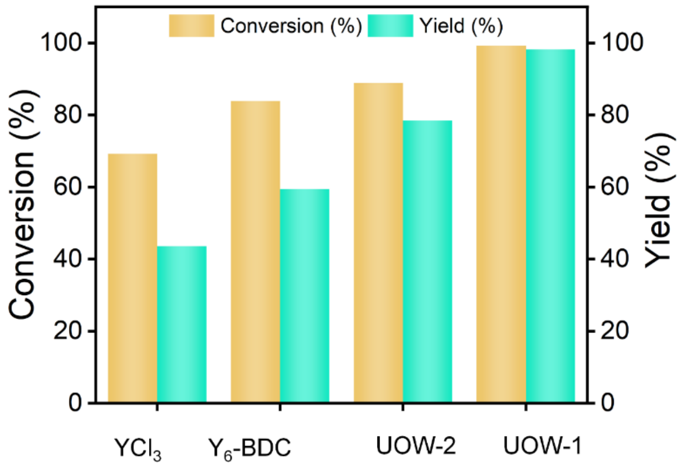

Figure S9. Screening of different yttrium-based catalysts for the cycloaddition of epichlorohydrin and CO_2 at 80 °C for 6 h. The YCl_3 is chosen as Y^{3+} source to demonstrate that the high-surface area MOF structure plays a key role in the efficiency of the catalytic reactions and the presence of only Y^{3+} is not sufficient for high activities.

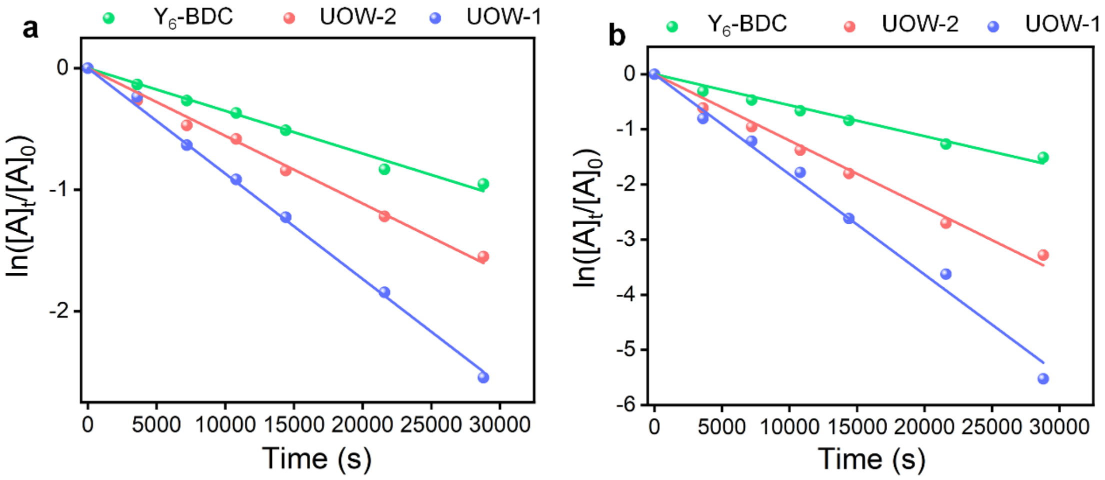

Figure S10. Reaction kinetics semi-logarithmic plots for all three catalysts at (a) 40 °C and (b) 60 °C.

Table S10. Catalytic comparison of MOF-based catalyst used in CO_2 cycloaddition reaction with epichlorohydrin as the model substrate

Catalyst	Ligand	Amt. catalyst (mg)	Amt. co-catalyst (mg)	Temp (ºC) Time (h)	Conversion (%)	Yield (%)	Ref.
**Cu PW MOF**	Octamethyl 5,5',5'',5'''-((methane tetrayl tetrakis(benzene-4,1-diyl)) tetrakis (1H1,2,3-triazole-4,1diyl))tetraiso Phthalate: **H_8L1**	15.6	650	RT<br>48	85	56	2
**MOF-Zn-1**	2,5-Thiophene dicarboxylic acid: **TFDCA**<br>Melamine: **MA**	100	100	80<br>3	-	63	3
**TMU-63S**	Di aza hexadiene ligands: **4-bpdh**<br>*N*[1],*N*[3]-di(pyridine-4-yl) malonamide **:4-dpm**	5	5	50<br>32	72	~63.5	4
**Zn-MOF-184**	4,4′-(ethyne-1,2-diyl)bis(2-oxidobenzoate): **$EDOB^{4-}$**	1.2 mol% active site	1.5mol%	80<br>6	~100	~70	5
**ZnTCPP⊂(Br^-)Etim-UiO-66**	Imidazolium terephthalic acid **: Im-H_2BDC**	0.95 mol%	-	140<br>14	-	86	6
**FJI-H14**	2,5-di(1H1,2,4-triazol-1-yl)terephthalic acid: **H_2BTTA**	18	164	80<br>24	-	95	7
**$Zn_{0.75}Mg_{0.25}$-MOF-74**	2,5-dihydroxyterephthalic acid: **H_4dhta**	0.59 mol%	100	60<br>5	-	95	8
**rho-ZMOF**	4,5-Imidazoledicarboxylic acid : **H_3ImDC**	25	200	40<br>3	98	~96.6	9
**CSMCRI-13**	4,4',4''-tricarboxytriphenylamine: **H_3TCA**<br>2,2′-Bipyridine **: bpy**	0.14 mol%	0.24 mol%	70<br>6	-	97	10
**MIL-101-IP**	1,4-benzenedicarboxylic acid: **BDC**	50	-	50<br>68	-	99	11

**Au@MOF**	2,6-bis(2′,5′-dicarboxylphenyl)pyridine: **H_4L'**	0.1	645	80<br>8	-	99	12
**UNM-6-Br**	Tetra butyl ammonium Tetrakis(4- pyridine tetra fluorophenyl) borate	50		115<br>72	95	-	13
**Y-TCPP**	2,3,5,6-tetrakis(4-carboxyphenyl)pyrazine: **H_4TCPP**	10	119.28	100<br>4	99	-	14
**gea-MOF-1**	2-fluorobenzoic acid	60	50	120<br>6	89	-	15
**PCN-224(Co)-BPDC-CH2NBu3 Br**	H_4TCPP,4,4-biphenyl dicarboxylic acid: **H_2BPDC**, benzoic acid	0.02 mmol based on Co	~23	100<br>4	90	-	16
**UOW-1**	2,5-furan dicarboxylate: **FDC** (renewable feedstock)	50	50	80<br>6	99.7	98.8	**This work**

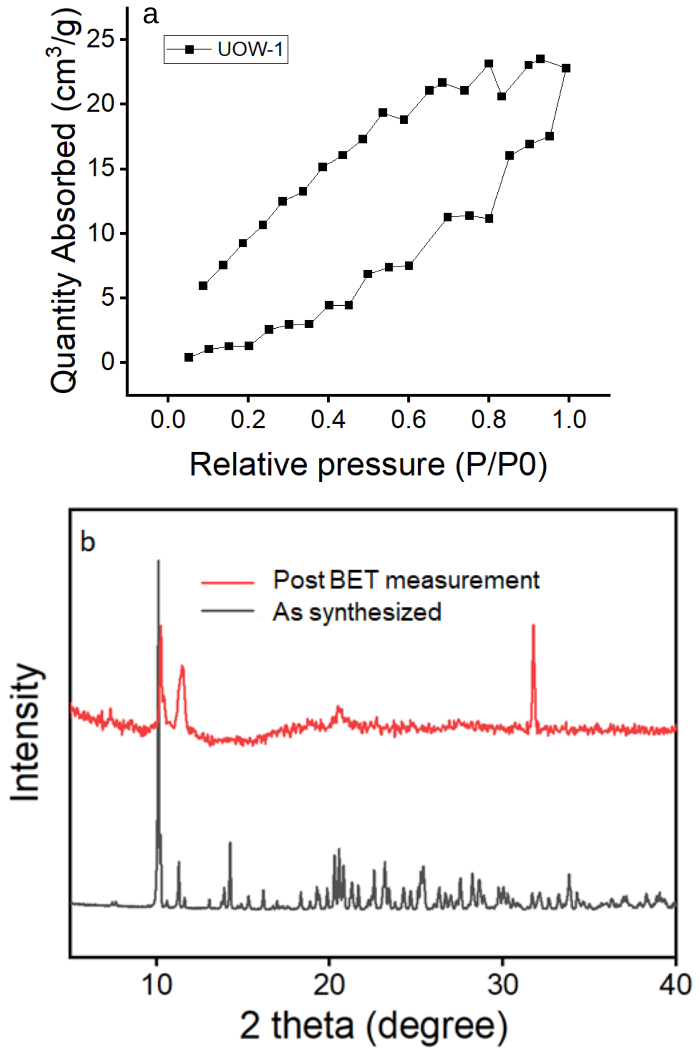

Figure S11: (a) Attempted nitrogen adsorption isotherm measurement of UOW-1 after activation at 120 °C for 6 hours, and (b) powder XRD of the material before and after.

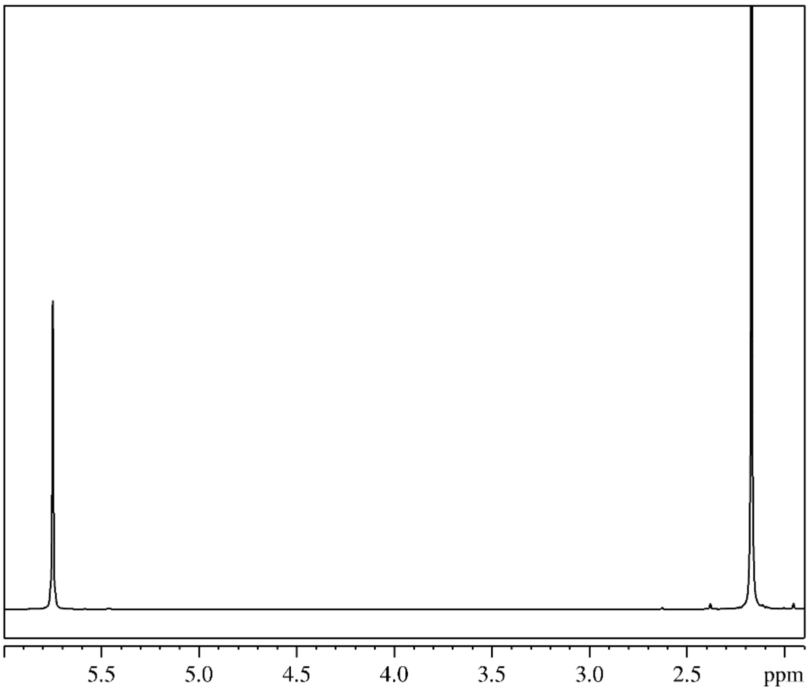

Figure S12: ^{1}H NMR of a solution of digested UOW-1 post catalysis showing absence of any substrate or product molecules with the pores.

Supporting References

(1) Du, X.; Fan, R.; Qiang, L.; Wang, P.; Song, Y.; Xing, K.; Zheng, X.; Yang, Y. Encapsulation and Sensitization of Ln^{+3} Within Indium Metal–Organic Frameworks for Ratiometric Eu^{+3} Sensing and Linear Dependence of White-Light Emission. *Cryst. Growth Des.* **2017**, *17*, 2746-2756.

(2) Li, P.; Wang, X.; Liu, J.; Lim, J.; Zou, R.; Zhao, Y. A Triazole-Containing Metal–Organic Framework as a Highly Effective and Substrate Size-Dependent Catalyst for CO_2 Conversion. *.J Am. Chem. Soc.* **2016**, *138*, 2142-2145.

(3) Lan, J.; Liu, M.; Lu, X.; Zhang, X.; Sun, J. Novel 3D Nitrogen-Rich Metal Organic Framework for Highly Efficient CO_2 Adsorption and Catalytic Conversion to Cyclic Carbonates Under Ambient Temperature. *ACS Sustain. Chem. Eng.* **2018**, *6*, 8727-8735.

(4) Gharib, M.; Esrafili, L.; Morsali, A.; Vande Velde, C.; Guo, Z.; Junk, P. Effective Dual-Functional Metal–Organic Framework (DF-MOF) as a Catalyst for the Solvent-Free Cycloaddition Reaction. *Inorganic Chemistry* **2022**, *61*, 6725-6732.

(5) Tran, Y.; Nguyen, P.; Luong, Q.; Nguyen, K. Series Of M-MOF-184 (M = Mg, Co, Ni, Zn, Cu, Fe) Metal–Organic Frameworks for Catalysis Cycloaddition of CO_2. *Inorg. Chem.* **2020**, *59*, 16747-16759.

(6) Liang, J.; Xie, Y.; Wu, Q.; Wang, X.; Liu, T.; Li, H.; Huang, Y.; Cao, R. Zinc Porphyrin/Imidazolium Integrated Multivariate Zirconium Metal–Organic Frameworks for Transformation of CO_2 Into Cyclic Carbonates. *Inorg. Chem.* **2018**, *57*, 2584-2593.

(7) Liang, L.; Liu, C.; Jiang, F.; Chen, Q.; Zhang, L.; Xue, H.; Jiang, H.; Qian, J.; Yuan, D.; Hong, M. Carbon Dioxide Capture and Conversion by an Acid-Base Resistant Metal-Organic Framework. *Nat. Commun.* **2017**, *8*.

(8) Gao, Z.; Liang, L.; Zhang, X.; Xu, P.; Sun, J. Facile One-Pot Synthesis of Zn/Mg-MOF-74 with Unsaturated Coordination Metal Centers for Efficient CO_2 Adsorption and Conversion to Cyclic Carbonates. *ACS Appl. Mater. Interfaces* **2021**, *13*, 61334-61345.

(9) Zhang, S.; Jang, M.; Lee, J.; Puthiaraj, P.; Ahn, W. Zeolite-Like Metal Organic Framework (ZMOF) with a rho Topology for a CO_2 Cycloaddition to Epoxides. *ACS Sustain. Chem. Eng.* **2020**, *8*, 7078-7086.

(10) Seal, N.; Neogi, S. Intrinsic-Unsaturation-Enriched Biporous and Chemorobust Cu(II) Framework for Efficient Catalytic CO_2 Fixation and Pore-Fitting Actuated Size-Exclusive Hantzsch Condensation with Mechanistic Validation. *ACS Appl. Mater. Interfaces* **2021**, *13*, 55123-55135.

(11) Aguila, B.; Sun, Q.; Wang, X.; O'Rourke, E.; Al-Enizi, A.; Nafady, A.; Ma, S. Lower Activation Energy for Catalytic Reactions through Host–Guest Cooperation within Metal–Organic Frameworks. *Angew. Chem. Int. Ed.* **2018**, *57*, 10107-10111.

(12) Wu, Y.; Yang, G.; Cheng, S.; Qian, J.; Fan, D.; Wang, Y. Facile Incorporation of Au Nanoparticles into an Unusual Twofold Entangled Zn(II)-MOF with Nanocages for Highly Efficient CO_2 Fixation under Mild Conditions. *ACS Appl. Mater. Interfaces* **2019**, *11*, 47437-47445.

(13) Thapa, S.; Meng, L.; Hettiarachchi, E.; Bader, Y.; Dickie, D.; Rubasinghege, G.; Ivanov, S.; Vreeland, E.; Qin, Y. Charge-Separated and Lewis Paired Metal–Organic Framework for Anion Exchange and CO_2 Chemical Fixation. *Chem. Eur. J.* **2020**, *26*, 13788-13791.

(14) Ye, Y.; Ge, B.; Meng, X.; Liu, Y.; Wang, S.; Song, X.; Liang, Z. An Yttrium-Organic Framework Based on a Hexagonal Prism Second Building Unit for Luminescent Sensing of Antibiotics and Highly Effective CO_2 Fixation. *Inorg. Chem. Front.* **2022**, *9*, 391-400.

(15) Guillerm, V.; Weseliński, Ł.; Belmabkhout, Y.; Cairns, A.; D'Elia, V.; Wojtas, Ł.; Adil, K.; Eddaoudi, M. Discovery and Introduction of a (3,18)-Connected Net as an Ideal Blueprint for the Design of Metal–Organic Frameworks. *Nat. Chem.* **2014**, *6*, 673-680.

(16) Zhang, L.; Yuan, S.; Fan, W.; Pang, J.; Li, F.; Guo, B.; Zhang, P.; Sun, D.; Zhou, H. Cooperative Sieving and Functionalization of Zr Metal–Organic Frameworks through Insertion and Post-Modification of Auxiliary Linkers. *ACS ACS Appl. Mater. Interfaces* **2019**, *11*, 22390-22397.
